# Functional outcomes post-stroke: Evidence from private rehabilitation facilities in South Africa between 2006 and 2023

**DOI:** 10.4102/hsag.v31i0.3174

**Published:** 2026-05-20

**Authors:** LeeAnne Masilela, Hendrik Loubser, Nicola Plastow, Daleen Casteleijn, Judith Bruce

**Affiliations:** 1 Department of Epidemiology and Biostatistics, Faculty of Health Sciences, University of the Witwatersrand, Johannesburg, South Africa; 2 Department of Nursing Education, Faculty of Health Sciences, University of the Witwatersrand, Johannesburg, South Africa; 3 The South African Database for Functional Medicine, Cape Town, South Africa; 4 Department of Occupational Therapy, Faculty of Health Sciences, Stellenbosch University, Cape Town, South Africa; 5 Department of Occupational Therapy, Faculty of Health Sciences, University of Pretoria, Pretoria, South Africa

**Keywords:** stroke, rehabilitation, functional outcomes, activities of daily living, BETA scale

## Abstract

**Background:**

Effective rehabilitation is crucial for stroke survivors to regain functional independence and reduce the healthcare burden associated with disability and hospital readmission.

**Aim:**

To evaluate the functional outcomes in patients with stroke at private rehabilitation facilities between 2006 and 2023.

**Setting:**

This was a retrospective cohort study of 9010 patients, post-stroke, at private rehabilitation facilities registered with the South African Board of Healthcare Funders.

**Methods:**

The 7-point BETA scale was used to measure inpatients’ ability to perform Activities of Daily Living (ADL) in real time. Secondary analysis was conducted on ADL data sets (*N* = 9010) from a purposive sample of private rehabilitation facilities (*n* = 47). Descriptive statistics were used to determine the median functional scores of stroke patients measured by the BETA scale. Linear regression was used to compare functional gain scores by baseline patient demographics, and logistic regression for the association between functional deterioration and patient factors, and to predict risk.

**Results:**

Functional outcomes showed limited improvement; 40.8% of inpatients experienced deterioration, 19.5% showed no functional gain, 31.9% showed modest gain, and a small minority (< 0.5%) achieved substantial functional gains. Predictors of functional outcomes included age, gender, racial demographics, facility type and stroke characteristics.

**Conclusion:**

The findings indicate suboptimal functional outcomes in patients, post-stroke, at private rehabilitation facilities. There is a need for targeted improvements in rehabilitation practices and policy changes to enhance post-stroke functional outcomes.

**Contribution:**

This study provides longitudinal perspectives of patients’ functional outcomes post-stroke in private rehabilitation facilities and an evidence base for informing rehabilitation practice improvements.

## Introduction

Globally, stroke remains an enormous health burden as one of the leading causes of morbidity and mortality, with ischaemic stroke accounting for 68% of cases (Markus 2023; Saceleanu et al. 2023). The Global Burden of Disease Study 2021 showed that stroke was the third most common cause of death (7.3 million deaths) after ischaemic heart disease and coronavirus disease 2019 (COVID-19), and the fourth most common cause of DALYs (Disability-Adjusted Life Years) (Feigin et al. 2024). Stroke often results in significant disability; it is a leading cause of acquired, permanent disability worldwide (Grefkes & Fink 2020; Rejnö et al. 2019), affecting patients’ physical and cognitive abilities and limiting their capacity to perform activities of daily living (ADLs) (Elendu et al. 2023; Shao et al. 2023); 89% of global stroke deaths and disability combined occur in low-to middle-income countries (World Stroke Organization 2022).

The burden of stroke is thus particularly pronounced in low- and middle-income countries where access to preventive and rehabilitative services may be constrained or below the recommended standards (Akinyemi et al. 2021; Urimubenshi et al. 2018). In sub-Saharan Africa, there has been a dramatic increase in hospital admissions of patients suffering a stroke with high in-hospital and post-discharge mortality rates, and a 3-year mortality rate greater than 80% (Akinyemi et al. 2021; Walker 2023). In South Africa, stroke is the second highest cause of mortality and disability (Matizirofa & Chikobvu 2021; Tribelhorn, Motara & Lewis 2021). Stroke accounts for over 25 000 deaths annually and 95 000 years lived with disability, which hampers socioeconomic development in South Africa (Ranganai & Matizirofa 2020). There is a significant economic burden in South Africa, which includes direct medical costs and other costs associated with stroke mortality and disability. Comorbidities such as diabetes and hypertension, which are highly prevalent in South Africa, further increase stroke-related costs (Ranganai & Matizirofa 2020). Effective rehabilitation is thus crucial for enabling stroke survivors to regain functional independence, improve their quality of life, and in the process, reduce the long-term healthcare burden associated with disability and hospital readmissions.

The functional independence of a patient post-stroke, particularly at the point of discharge, is arguably one of the most valuable predictors of hospital readmission within 1 year (Mauti et al. 2025). Hospital readmission, also characterised as the revolving door phenomenon, not only doubles the cost of care for patients treated in post-acute care but is also associated with a higher risk of dying in older adults living in communities. On the back of Porter’s (2010) advocacy for value-based care over a decade ago, several studies identified a common modifiable risk factor and concluded that predictive models, which consider comorbidities without accounting for functional status, have inferior performance value. Functional ability at discharge is thus seen as a reliable indicator of a stroke survivor’s ongoing needs and forms the basis of discharge planning and non-institutionalised care. With value-based healthcare outcomes in mind, this study focused on functional outcomes at the point of discharge of patients, post-stroke.

### Challenges in stroke rehabilitation in South Africa

Stroke rehabilitation is integral to enhancing recovery and restoring patients’ ability to perform ADLs, which are often compromised following a stroke. Effective rehabilitation can reduce the burden on caregivers, improve patients’ functional status and lower healthcare costs by decreasing the likelihood of readmission and long-term institutional care (Langhorne, Bernhardt & Kwakkel 2011).

However, the challenges of stroke rehabilitation in private facilities are threefold. Firstly, rehabilitation case management and care coordination are driven from a funder or medical insurance perspective with a focus on cost containment and cost savings (Medford-Davis et al. 2016). Consequently, value-based stroke care that occurs at institutional level may be thwarted if the concept of value is defined by the health outcomes achieved in monetary currency (Porter & Kaplan 2016; Zafar et al. 2020). This, in turn, may lead to poor rehabilitation outcomes and suboptimal integration into communities of stroke survivors, with little funds allocated to prevent the readmission of discharged patients. Secondly, the role of nurses in stroke rehabilitation is poorly defined, including their responsibilities in evaluating functional outcomes using measures such as the BETA scale. Discharge planning, especially for early supported discharge, is a key nursing responsibility, and with appropriate post-registration training in rehabilitation (Byrne et al. 2025; Zhao et al. 2024), nurses can play a significant role within multidisciplinary rehabilitation teams. Thirdly, social workers and occupational therapists are integral to the discharge, return to work and reintegration into communities of stroke survivors. Although they are responsible for developing seamless community integration pathways and support systems for patients with functional loss, social workers are neither acknowledged nor supported by the funder case management system.

In the public sector too, rehabilitation capacity in South Africa is severely constrained relative to the population’s needs. As recently as 2 years ago, the public sector had 286 rehabilitation beds designated for long-term stroke care for adult patients – a fraction of what is needed given the high incidence of stroke (Louw et al. 2023). Rehabilitation facilities are generally indiscriminate and may include any setting outside of acute care, e.g. step-down units, general wards, outpatient departments (OPD’s), elderly care, designated rehabilitation units, etc. However, most of these facilities are in the private sector, making them inaccessible to large parts of the indigent population. Furthermore, rehabilitation authorisation decisions such as triage, follow-up and discharge are guided by clinical data interpreted by nursing case managers – a process that is discipline-specific rather than comprehensive functional assessments such as ADLs. This can lead to suboptimal care pathways and hinder reasonable value-based rehabilitation outcomes for stroke patients.

To address the need for more structured outcomes data in rehabilitation, the South African Database for Functional Medicine (SADFM) was developed. The SADFM collects real-time data on patient functional outcomes over time and settings, using a suite of routine outcome measures. One of these outcome measures is the 18-item BETA scale (Loubser, Casteleijn & Bruce 2015).

### Study aim and objectives

This study aimed to evaluate functional outcomes in patients with stroke at private rehabilitation facilities in South Africa over an 18-year period, using the BETA scale data associated with functional gain and deterioration. The objectives were to: Establish baseline demographic and functional variables of patients admitted with stroke to private facilities providing rehabilitation services; determine the distribution of functional gain and/or deterioration between the date of discharge and admission; and investigate the association between demographic and functional variables.

## Research methods and design

Following a retrospective cohort study design, secondary analysis was conducted on the ADL data of inpatients diagnosed with stroke.

### Population and sample

Patients diagnosed with stroke in private rehabilitation facilities nationally were the target population (*N* = 9010); the study population were the electronic records in SADFM, of stroke patients admitted to these facilities between January 2006 and May 2023. No other selection criteria were applied, and the total number of records (*n* = 9010) was included in the analysis.

Rehabilitation facilities were purposively selected and included if they were registered with the South African Board of Healthcare Funders and had a practice number (*n* = 47). Facilities offering rehabilitation services included dedicated sub-acute care facilities (86.25%), general wards in hospitals (12.82%) and the remainder (< 1%) received rehabilitation services at home (home-based care), elderly care facilities or as outpatients.

### Data source and data collection tool

The data source for secondary analysis was the SADFM electronic records, which comprised patient ADL data. Using direct observation, these retrospective data were collected and entered by nurses, therapists and caregivers who were trained, accredited and tested for competence (set at 80%), and reliability in using the 7-point BETA scale. Although BETA scale data were routinely collected during the length of stay, BETA scores at only two key time points, namely, on admission to the rehabilitation facility and at discharge, were applicable in this study.

The BETA scale is embedded in the nursing process and used as a routine observational measure of the patient’s functional ability to execute their ADLs in motor and cognitive domains, rated between one and seven. It comprises 18 items: 13 items measure motor ability associated with self-care (eating, grooming, bathing, dressing upper and lower body and toileting), sphincter control (bowel and bladder control) and mobility (transfers from bed or chair, toilet and bath, walking or wheelchair ambulation and stairs); five items measure cognitive abilities, including comprehension, expression, problem solving, social interaction and memory.

The analysis included demographic, clinical and facility-related variables available in the database to determine their potential influence on functional outcomes. Demographic factors were gender, race, age on admission, length of stay and the only clinical variable, stroke type or stroke description. Stroke description documented by healthcare staff included stroke-bilateral involvement, stroke-left body involvement, stroke-right body involvement, stroke-no paresis and stroke-unspecified.

### Data analysis

Secondary data analysis was performed using Stata version 16. Descriptive statistics summarised demographic and clinical characteristics of the patient cohort, with parametric or nonparametric methods applied based on the data distribution. Functional outcomes were assessed by calculating the percentage change in BETA scores between admission and discharge. The percentage was derived by dividing the BETA score at each time point by the total possible score, then multiplying by 100 to standardise the measure. Patients were classified into eight distinct outcome groups: < 0% = functional deterioration, 0% = no gain, 1% – 20% = gain, 20% – 40% gain, 40% – 60% gain, 60% – 80% gain, 80% – 85% gain and 85% – 100% gain. This classification allowed for a view of the range of functional gains and losses experienced by patients during rehabilitation.

A trend analysis was conducted to assess the distribution of functional gain scores (any positive increase in BETA score percentage from admission to discharge), identifying any changes in rehabilitation outcomes over time. Linear regression models were computed to evaluate the predictors of functional gain as a continuous outcome, with functional gain defined as a positive increase in BETA scores from admission to discharge. Logistic regression analysis was performed with binary outcomes for functional gain (functional gain = yes) and functional deterioration (functional gain = no), incorporating all available demographic and facility-related variables as predictors. Functional deterioration was defined as any decrease in BETA scores from admission to discharge. This approach allowed for the identification of key factors associated with both positive and negative functional outcomes, providing insight into the influence of patient and facility characteristics on rehabilitation effectiveness. Missing data were reported in the respective tables. A level of significance of *p* < 0.05 was used.

### Ethical considerations

Ethical clearance to conduct this study was obtained from the University of the Witwatersrand Human Research Ethics Committee (Medical). The ethical clearance number is M231140 M240802-A0002. The BETA score data used for this study had been previously collected and secured in the SADFM database. A facility licence was issued by the SADFM to participating rehabilitation facilities to collect the BETA scale data as patient-based evidence of their outcome performance achieved. To ensure confidentiality and anonymity, data collectors signed a compliance agreement in terms of the Protection of Personal Information Act (South Africa 2013) and received a personalised, unique login and password to enter patient scores into the SADFM database. All data columns with patient and facility identifiable data were encrypted by the SADFM technical team before they were made available to the researchers. The SADFM and the University of the Witwatersrand have signed a collaboration agreement, whereby de-identified data sets are accessible for further analyses by researchers.

## Results

The sample (*N* = 9010) comprised 50.1% female patients (*n* = 4513), with a diverse racial representation: 44.1% White; 26.2% Black; 10.3% Mixed race; 3.3% Asian and 16.1% categorised as ‘Other’. The median age of participants was 66 years (interquartile range [IQR] 54–77), and the median length of stay was 18 days (IQR 15–21). Most patients (86.2%) were admitted to sub-acute facilities, with general wards accounting for 12.7% of admissions. Strokes with left body involvement (*n* = 2551; 28.31%) and right body involvement (*n* = 2223; 24.67%) were almost equally distributed. Most of the remaining patients were classified as ‘Stroke-unspecified’ (Table 1).

**TABLE 1:** Baseline characteristics of stroke patients admitted to rehabilitation facilities between 2006 and 2023 (*N* = 9010).

Baseline characteristics	*n*	%
**Gender**		
Female	4513	50.09
Male	4497	49.91
**Race**		
Asian people	294	3.26
Black people	2363	26.23
Mixed-race people	929	10.31
White people	3973	44.10
Other	1450	16.09
Missing	1	0.01
**Rehabilitation facility**		
General ward	1155	12.82
Home-based care	36	0.40
Elderly care	14	0.16
OPD	34	0.38
Sub-acute care	7771	86.25
**Stroke description**		
Stroke-bilateral involvement	425	4.72
Stroke-left body involvement	2551	28.31
Stroke-no paresis	171	1.90
Stroke-unspecified	3640	40.40
Stroke-right body involvement	2223	24.67

Note: Median Age (years) = 66, IQR = 23; Median Length of stay (days) = 18, IQR = 15–21.

OPD, outpatient department; IQR, interquartile range.

### Functional scores and outcomes

Table 2 shows the functional outcomes assessed using the BETA scale. On admission, the median BETA score was 48% (IQR: 28%–71%), indicating substantial functional limitations amongst patients upon entering rehabilitation. At discharge, the median BETA score was 47% (IQR: 27%–70%), reflecting deterioration in functional ability over the rehabilitation period. Cognitive function and motor function scores on admission and discharge showed negative change or no change, respectively, underscoring poor functional gains overall.

**TABLE 2:** Total BETA functional ability scores (in %) of stroke patients (*N* = 9010).

Functional ability	Percentage				Skewness	Kurtosis
	Median	IQR	Mean	s.d.		
Admission score	48	28–71	50.1	24.6	0.23	1.88
Cognitive function on admission	58	35–80	57.5	27.5	0.06	1.79
Motor function on admission	41	22–69	46.6	26.1	0.41	1.90
Discharge score	47	27–70	49.5	24.6	0.24	1.87
Cognitive function at discharge	55	32–80	56.8	27.6	0.10	1.79
Motor function at discharge	41	22–69	46.6	26.1	0.43	1.92

s.d., standard deviation; IQR, interquartile range.

### Functional gain distribution

**FIGURE 1:**
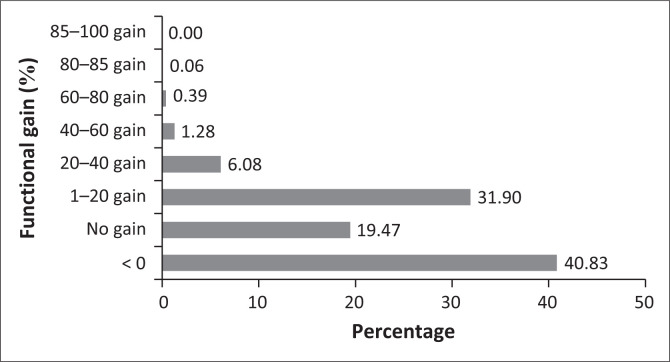
Distribution of functional gain score of admitted stroke patients, 2006–2023.

Figure 1 illustrates the distribution of functional gain amongst patients, post-stroke, during rehabilitation. A substantial proportion of patients (40.83%) experienced a decline in functional ability, with scores decreasing from admission to discharge. An additional 19.47% of patients showed no functional gain, resulting in a combined 60.3% of patients who either deteriorated or showed no gains at all. Of those who achieved positive gains, the majority (31.9%) recorded only modest improvements in the 1% – 20% range. A smaller fraction of patients reached higher levels of functional gain: 6.08% achieved functional gains between 20% and 40%, 1.28% between 40% and 60% and 0.39% between 60% and 80%, whilst gains above 80% were extremely rare.

**FIGURE 2:**
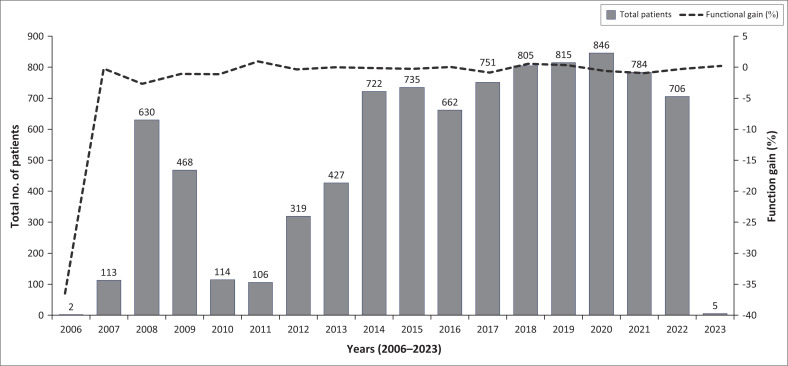
Distribution of functional gain scores of patients admitted with stroke over time.

Figure 2 shows the number of patients with stroke admitted to rehabilitation facilities between 2006 and 2023, alongside the trend in functional gain percentages over time. Despite fluctuations in the number of patients admitted, the functional gain percentages remained relatively stable, indicating a consistent, limited improvement in functional recovery across the years.

### Predictors of functional gain and deterioration

Linear regression analysis (Table 3) identified several key predictors of functional gain. Female patients showed higher mean gain scores compared to males, suggesting gender-based differences in functional outcomes; these differences were statistically significant (*p* = 0.006). Racial disparities were also observed, with Black, Mixed-race and White patients exhibiting significantly lower functional gains than those in the ‘Other’ category, with Black patients having the largest negative association (*p* < 0.001). The type of facility influenced functional outcomes; patients receiving rehabilitation whilst in general wards showed lower functional gains compared to those in sub-acute facilities (*p* = 0.001). The stroke involvement or description category also impacted outcomes, with patients who suffered a left-sided stroke demonstrating higher functional gains (*p* = 0.005). Higher baseline motor function scores at admission were positively correlated with functional gains (*p* < 0.001), highlighting the importance of initial motor abilities for recovery potential.

**TABLE 3:** Linear regression univariable and multivariable analysis of factors associated with functional gain score in stroke patients (*N* = 9010).

Variable	Univariable analysis			Multivariable analysis		
	Coef.	95% CI	*p*-value	Coef.	95% CI	*p*-value
**Gender**						
Female	0.880	0.200–1.560	0.011	1.1900	0.340–2.040	0.006
Male	1.000	-	1.000	1.0000	-	1.000
**Race**						
Asian people	0.580	−1.490–2.660	0.581	−0.5900	−5.000–3.830	0.795
Black people	0.720	−10.350–1.810	0.183	−2.4000	−3.720 to -1.070	< 0.001
Mixed-race people	−0.730	−2.090–0.630	0.290	−2.1100	−3.550 to -0.670	0.004
White people	0.730	−0.260–1.730	0.148	−1.3300	−2.470 to -0.180	0.023
Other	1.000	-	1.000	1.0000	-	1.000
**Rehabilitation facility**						
General ward	0.300	−0.720–1.330	0.558	−3.8300	−6.080 to -1.570	0.001
Home Based	1.720	−3.620–7.050	0.528	−1.4600	−7.570–4.650	0.640
Elderly care	−2.080	−10.740–6.580	0.638	1.6200	−11.300–14.530	0.806
OPD	3.270	−2.380–8.920	0.257	−0.6700	−18.920–17.580	0.943
Sub-Acute	1.000	-	1.000	1.0000	-	1.000
**Stroke description**						
Stroke-bilateral involvement	−0.150	−1.810–1.510	0.861	0.6100	−2.020–3.230	0.651
Stroke-left body involvement	−0.660	−1.500–0.170	0.120	1.5300	0.450–2.600	0.005
Stroke-no paresis	−0.500	−3.030–2.030	0.699	−6.0300	−9.330 to -2.730	< 0.001
Stroke-unspecified	1.000	-	1.000	1.0000	-	1.000
Stroke-right body involvement	−1.040	−1.910 to -0.170	0.019	1.5300	0.450–2.600	0.005
Age	0.010	−0.010–0.030	0.582	−6.0300	−9.340 to -2.730	< 0.001
Length of stay	0.003	−0.004 to -0.002	< 0.001	−0.0001	−0.002 to -0.001	< 0.001
Admission cognitive function score	−0.150	−0.170 to -0.150	< 0.001	−0.4400	−0.460 to -0.420	< 0.001
Admission motor function score	0.210	0.200–0.220	< 0.001	0.5500	0.530–0.570	< 0.001

OPD, outpatient department; CI, confidence interval; Coef, coefficient.

Logistic regression analysis (Table 4) identified predictors of functional deterioration; regression analysis was limited to 7298 patients who experienced either gain or deterioration; patients with 0% gain were removed from the analysis (*n* = 1712). Black and White patients had higher odds of deterioration compared to other racial groups (*p* < 0.001). Facility type was also a significant factor as admission to general wards increased the likelihood of deterioration (*p* = 0.001), further supporting the potential benefit of sub-acute care facilities or similar forms of transitional care in promoting recovery. Stroke clinical characteristics that influenced deterioration risks included patients who had right-sided body stroke involvement and those without paresis, showing higher odds of deterioration. Conversely, higher motor scores on admission were associated with a reduced risk of deterioration (*p* < 0.001), reinforcing the impact of initial functional status on recovery outcomes. Additionally, cognitive scores on admission were significantly associated with an increased risk of functional deterioration at discharge (*p* < 0.001). This suggests that the higher the BETA cognitive functioning of stroke patients on admission, the more likely their total BETA scores will show a decline by discharge.

**TABLE 4:** Logistic regression univariable and multivariable analysis of factors associated with functional deterioration in stroke patients (*n* = 7298).

Variable	Univariable analysis			Multivariable analysis		
	Coef.	95% CI	*p*-value	Coef.	95% CI	*p*-value
**Gender**						
Female	0.890	0.8100–0.9600	0.005	0.91	0.78–1.05	0.200
Male	1.000	-	1.000	1.00	-	1.000
**Race**						
Asian people	0.860	0.6600–1.1100	0.248	1.63	0.75–3.25	0.214
Black people	1.070	0.9400–1.2200	0.313	1.65	1.30–2.08	< 0.001
Mixed-race people	1.010	0.8500–1.1900	0.954	1.13	0.87–1.46	0.366
White people	1.050	0.9300–1.1900	0.425	1.44	1.18–1.46	< 0.001
Other	1.000	-	1.000	1.00	-	1.000
**Rehabilitation facility**						
General ward	1.230	1.0800–1.3900	0.001	1.88	1.28–2.76	0.001
Home Based	0.470	0.2200–0.9900	0.047	1.77	0.66–4.70	0.255
Old-age related	5.330	1.4900–19.1200	0.010	1.56	0.20–12.14	0.673
OPD	0.540	0.2500–1.1700	0.121	-	-	-
Sub-Acute	1.000	-	1.000	1.00	-	1.000
**Stroke description**						
Stroke-bilateral involvement	1.000	0.8100–1.2200	0.973	1.03	0.66–1.60	0.906
Stroke-left body involvement	1.130	1.0200–1.2500	0.017	0.79	0.66–0.96	0.015
Stroke-no paresis	1.080	0.7900–1.4800	0.619	1.89	1.08–3.27	0.024
Stroke-unspecified	1.000	-	1.000	1.00	-	1.000
Stroke-right body involvement	1.150	1.0300–1.2800	0.010	1.03	0.86–1.26	0.694
Age	0.990	0.9920–0.9980	0.001	0.99	0.98–1.00	< 0.001
Length of stay	1.001	1.0001–1.0002	< 0.001	1.00	0.99–1.04	0.127
Time from onset to admission	1.000	0.9900–1.0010	0.354	1.00	0.99–1.01	0.437
Admission cognitive function score	1.020	1.0100–1.0200	< 0.001	1.04	1.04–1.05	< 0.001
Admission motor function score	0.990	0.9800–0.9900	< 0.001	0.96	0.95–0.96	< 0.001

OPD, outpatient department; CI, confidence interval; Coef, coefficient.

## Discussion

This study provides a detailed analysis of post-stroke functional outcomes in private rehabilitation facilities in South Africa. It reveals unsatisfactory functional outcomes of stroke survivors before being reintegrated into communities. This result aligns with the findings of a narrative review of stroke research in South Africa that stroke survivors have poor functional ability at discharge from the hospital, amidst other socioeconomic factors (Ntsiea 2019). We found that nearly 60% of patients showed either no improvement or a decline in functional ability. They also required a longer stay in the healthcare facility, with the potential for increased costs to the health insurance industry. Additionally, prolonged inpatient rehabilitation creates facility fatigue syndrome, well known to give rise to deconditioning, including muscular wasting and decline at a rate of 2% – 5% per day (McDonald et al. 2021). This ‘pyjama paralysis’ in facilities may contribute to worsening functional outcomes despite ongoing rehabilitation interventions aimed at achieving the direct opposite outcome. In general, motor and cognitive functional status that signals high dependence at discharge has high odds of unplanned rehospitalisation (Middleton et al. 2016). These findings underscore the need to improve functional status from admission to discharge to secure value-based delivery of rehabilitation outcomes in private, post-acute rehabilitation facilities and services.

From a predictability perspective for at-risk patients with stroke, regression analyses showed that factors such as gender, race, facility type, stroke characteristics, and baseline functional scores are significant predictors of functional outcomes. When comparing outcome and value-based performance between the rehabilitation services rendered in sub-acute facilities and in hospital general wards, sub-acute care facilities were associated with better outcomes than general wards. This implies that specialised environments with greater access to rehabilitation may provide more supportive conditions for post-stroke care. However, even within sub-acute facilities, functional gains were generally modest, reflecting broader challenges in delivering value-based stroke rehabilitation in South Africa. This is an important consideration amidst mounting calls for dedicated stroke units (Pillay et al. 2023; SA-CSRG 2019), which are reportedly evolving across the healthcare systems in South Africa (Smythe et al. 2023; Van Niekerk et al. 2021).

The results further point to systemic challenges within South Africa’s private rehabilitation services, where outcomes appear to fall short of international standards. Globally, stroke rehabilitation is recognised as a critical component of post-stroke care, with numerous studies and reviews demonstrating the benefits of structured, multidisciplinary rehabilitation for improving functional recovery and quality of life (Langhorne et al. 2018; Ntsiea 2019; Winstein et al. 2016). However, despite the publication of the South African Stroke Rehabilitation Guidelines (SA-CSRG 2019), which aim to address local needs through contextualised protocols this study suggests that these standards may not have been fully implemented or may not adequately address the complex requirements of effective stroke rehabilitation. Factors contributing to poor outcomes may be patient-specific characteristics, including age accompanied by its innate comorbidities and initial motor function, which are significant predictors of post-stroke functional outcomes. The analysis confirmed the prediction that the higher the baseline motor scores on admission, the stronger the likelihood that the outcomes and value-based rehabilitation would be successful (Veerbeek et al. 2011). However, this prediction does not hold true for cognitive scores on admission to rehabilitation facilities. Patients with higher cognitive scores at baseline admission to rehabilitation facilities were more likely to experience cognitive functional decline at discharge. In a United Kingdom (UK) population-based study, it was found that 37% of stroke survivors either cognitively improved, deteriorated (30%) or remained unchanged (33%) during the first 3 months post-stroke with the risks of death, dependency, depression and institutionalisation (Obaid et al. 2020). Our finding was counterintuitive and is the focus of further investigation using measures with greater cognitive specificity.

Moreover, there are racial disparities in functional gains, with Black and Mixed-race patients experiencing lower improvements. This may reflect broader social determinants of health and access disparities at play even within the private healthcare sector, where patients likely face barriers related to cultural, socioeconomic or linguistic factors. A population-based study in the UK found that Black African and Caribbean patients had longer post-stroke survival rates but poorer functional outcomes, although these differences were not fully explained by sociodemographic, stroke-related and other clinical factors (Emmett et al. 2025). Poor functional outcomes were similar for Black and White patients in this study; however, Black patients were more likely to experience functional deterioration. As in Emmett et al.’s (2025) study, further research is required to explore the effect of socioeconomic, stroke- and other health-related factors.

Challenges persist in the availability and accessibility of comprehensive rehabilitation services in both public and private facilities. Despite being better resourced compared to public facilities, the private sector faces limitations, particularly in staffing specialised rehabilitation professionals, such as occupational therapists, speech therapists, physiotherapists and mental health practitioners. The importance of multidisciplinary care in stroke recovery is well-documented, with studies highlighting that access to coordinated rehabilitation teams leads to better functional outcomes, reduced disability (Langhorne et al. 2018; Winstein et al. 2016) and lower in-hospital mortality (Adeniji et al. 2023). In particular, the crucial role of nurses in comprehensive stroke rehabilitation and care, and the education and training needed to enhance stroke care by nurses, is well described (Camicia et al. 2021; Zhao et al. 2024). These findings indicate the necessity of universal and consistent stroke education for nurses to improve patient outcomes in stroke care from initial assessment through to rehabilitation and psychological support (Camicia et al. 2021).

This study demonstrates evidence of post-stroke functional deterioration in private rehabilitation services. Implementing evidence-based practices and adhering to standardised rehabilitation protocols that include task-specific training, intensive, coordinated therapy and person-centred care are essential to improve functional recovery (Jesus et al. 2016; Pollock et al. 2014). South Africa has committed itself to the WHO vision of achieving equitable, evidence-based rehabilitation for all by 2030 (Grimmer et al. 2020). Persistent barriers, such as insufficient training, resource constraints including stroke care infrastructure (Pillay et al. 2023), poor service integration and fragmented care (Halabi, Khalaf & Bani Hani 2021; Louw et al. 2023), may thwart this commitment and hinder the implementation of best practices.

### Limitations

This study has several limitations that should be considered when interpreting the findings. The SADFM, which primarily includes patients from private rehabilitation facilities, may limit the generalisability of results to the broader South African population, particularly those receiving care in public or rural settings. Whilst efforts were made to include diverse facility types within the private sector, findings may not fully represent the rehabilitation experiences of patients in resource-limited environments. Additionally, the analysis was based solely on functional scores recorded at admission and discharge, without follow-up data to assess long-term recovery outcomes. This limitation restricts insights into the sustainability of functional improvements and potential regression post-rehabilitation.

Although the BETA scale has been validated in clinical settings, it may not capture all aspects of functional recovery, especially subtle changes in ADL and quality of life. The decision to report functional scores as percentages rather than raw BETA scale scores was made to improve interpretability. However, this approach may not fully convey the clinical significance of changes in functional ability. Future studies should explore calibration methods to improve the interpretability of percentage-based functional scores and ensure consistency with rehabilitation benchmarks.

Confounding variables, such as socioeconomic status, comorbidities and differences in rehabilitation protocols across facilities, could not be fully accounted for in this study. Whilst regression models were used to adjust for key demographic and clinical factors (e.g. age, gender, stroke type and facility type), residual confounding variables remain a possibility.

Finally, patients’ length of stay posed a potential limitation, as longer periods of rehabilitation may have influenced functional outcomes. To minimise this impact, length of stay was included as an adjustment variable in the analysis; however, future studies should investigate how rehabilitation intensity, duration and facility-specific practices contribute to stroke recovery. Despite these limitations, the study provides valuable insights into stroke patients’ functional outcomes in private rehabilitation facilities, highlighting critical areas for improvement in rehabilitation practices and policy.

### Implications for policy and practice

Standardised rehabilitation guidelines grounded in evidence, and practical and applicable to local contexts, are key. Policymakers are required to focus on expanding access to multidisciplinary rehabilitation teams throughout the services, whilst developing and implementing policies that incentivise specialised staff availability in underserved areas.

Variation in recovery results across different facility types indicates that sub-acute care settings likely offer a better rehabilitation environment for stroke survivors. The healthcare system could see improved stroke outcomes through the development of sub-acute facilities and the adoption of these models in general wards. The implementation of such policy changes could serve public health interests because rehabilitation plays a vital role in both reducing disability and improving the quality of life amongst stroke survivors.

### Future research

Further studies are necessary to determine stroke rehabilitation results in the public sector and assess interventions that can improve recovery across both public and private facilities. The sustainability of functional recovery after discharge could be better understood through longitudinal research that tracks patient outcomes and highlights critical factors for long-term rehabilitation success. Qualitative research that examines patient and provider perspectives can reveal facilitators of and obstacles to successful rehabilitation and direct service improvements.

## Conclusion

In conclusion, private rehabilitation facilities studied exhibit significant effectiveness gaps in stroke rehabilitation, which necessitate service and practice improvements. Targeted interventions are essential, and uniform standards should be established across facilities to address factors impacting functional outcomes. Coordinated stroke care activities that optimise functional gains and minimise cognitive decline, preferably in designated units equipped and resourced for post-stroke care, are vital. Closing these gaps will lead to improved functional recovery whilst decreasing disability, enhancing the quality of life for stroke survivors and lowering hospital readmission rates.
